# Deciphering Tumor Response: The Role of Fluoro-18-d-Glucose Uptake in Evaluating Targeted Therapies with Tyrosine Kinase Inhibitors

**DOI:** 10.3390/jcm13113269

**Published:** 2024-05-31

**Authors:** Kalevi Kairemo, Mohamed Gouda, Hubert H. Chuang, Homer A. Macapinlac, Vivek Subbiah

**Affiliations:** 1 Department of Nuclear Medicine, The University of Texas MD Anderson Cancer Center, Houston, TX 77030, USA; 2 Department of Investigational Cancer Therapeutics, Division of Cancer Medicine, The University of Texas MD Anderson Cancer Center, Houston, TX 77030, USA; 3 Sarah Cannon Research Institute, Nashville, TN 37203, USA

**Keywords:** fluoro-18 deoxyglucose (18F-FLT), positron emission tomography/computerized tomography (PET/CT), molecular imaging, tyrosine kinase receptors, response evaluation, targeted therapy

## Abstract

**Background/Objectives**: The inhibitory effects of tyrosine kinase inhibitors (TKIs) on glucose uptake through their binding to human glucose transporter-1 (GLUT-1) have been well documented. Thus, our research aimed to explore the potential impact of various TKIs of GLUT-1 on the standard [^18^F]FDG-PET monitoring of tumor response in patients. **Methods**: To achieve this, we conducted an analysis on three patients who were undergoing treatment with different TKIs and harbored actionable alterations. Alongside the assessment of FDG data (including SUVmax, total lesion glycolysis (TLG), and metabolic tumor volume (MTV)), we also examined the changes in tumor sizes through follow-up [^18^F]FDG-PET/CT imaging. Notably, our patients harbored alterations in BRAFV600, RET, and c-KIT and exhibited positive responses to the targeted treatment. **Results**: Our analysis revealed that FDG data derived from SUVmax, TLG, and MTV offered quantifiable outcomes that were consistent with the measurements of tumor size. **Conclusions**: These findings lend support to the notion that the inhibition of GLUT-1, as a consequence of treatment efficacy, could be indirectly gauged through [^18^F] FDG-PET/CT imaging in cancer patients undergoing TKI therapy.

## 1. Introduction

As personalized medicine continues to reshape cancer treatment paradigms, the assessment of therapeutic responses becomes increasingly nuanced. Tyrosine kinase inhibitors (TKIs) have emerged as potent tools in targeting specific molecular pathways driving tumorigenesis. However, the efficacy of these targeted therapies necessitates meticulous monitoring to gauge treatment response accurately. 

**Figure 1:**
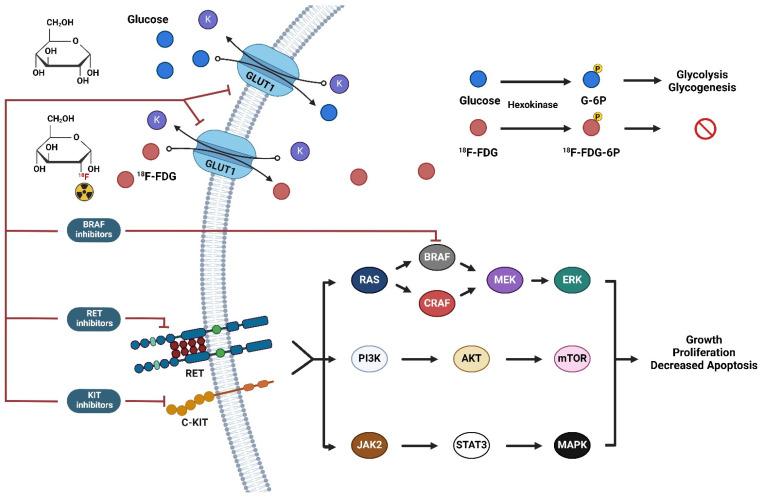
Schematic illustration of the transportation of deoxyglucose and 2-deoxy-2-[^18^F]fluoro-d-glucose ([^18^F]FDG) by the multifunctional transporter protein GLUT1 and their cellular metabolisms. The mechanism of action of some tyrosine kinase inhibitors (TKIs) (BRAF, RET, and ALK inhibitors) is presented in relation to possible inhibition by the glucose transporter protein GLUT1.

It is known that tyrosine kinase inhibitors reduce glucose uptake by binding to an exofacial site on human glucose transporter-1(GLUT-1), and the influence on [^18^F] FDG PET uptake has been studied experimentally [1,2]. The mechanism of action of some tyrosine kinase inhibitors and their relation to possible inhibition by the GLUT-1 transporters is shown in Figure 1.

Positron emission tomography (PET) imaging using 2-deoxy-2-[^18^F] fluoro-d-glucose ([^18^F] FDG) as an imaging agent plays a pivotal role in oncology, e.g., cancer diagnosis and staging. [^18^F] FDG is a marker of energy metabolism and cell proliferation, and it is routinely used in the clinic to evaluate patient responses to chemotherapy [3,4,5,6], and it can be used to monitor tumor growth. Treatment with some tyrosine kinase inhibitors (TKIs) may cause changes in blood glucose levels [2,7,8]. Additionally, the interaction of TKIs with amino acid residues at the glucose binding site may inhibit glucose uptake by GLUT-1 [1,2]. In this study, we propose that the inhibition of GLUT-1 by tyrosine kinase inhibitors (TKIs) may have significant implications for the evaluation of tumor response in patients undergoing TKI treatment. It is essential to monitor treatment effectiveness in order to make informed clinical decisions, ensuring that patients receive effective therapy while minimizing the risks associated with ineffective treatment and its side effects. Positron emission tomography (PET) is commonly utilized for monitoring treatment response, including the use of TKIs. In this analysis, we aim to investigate the potential impact of GLUT-1 inhibition by different TKIs on the routine monitoring of tumor response through [^18^F] FDG-PET imaging and explore the significance of FDG uptake in evaluating responses to targeted therapies with TKIs, exploring its potential as a biomarker for treatment efficacy and patient outcomes using some illustrative examples.

## 2. Methods and Patient Cases

In our investigation, we analyzed the medical documents of three individuals diagnosed with advanced malignancies who underwent FDG PET imaging while receiving targeted therapy with tyrosine inhibitors. This research adhered to the guidelines set forth by the MD Anderson Institutional Review Board (IRB, Houston, TX, USA). Given the retrospective nature of this chart review, the IRB exempted the need for consent. The patients in this study were enrolled in trials involving BRAF, RET, and c-KIT, which were accessible through our institution. Our study also ensured compliance with the Health Insurance Portability and Accountability Act. Each participant provided written informed consent prior to being enrolled in the relevant clinical trials.

Image analysis: All patients were analyzed with quantitative PET, utilizing standardized uptake values (SUV), metabolic tumor volume (MTV), and total lesion glycolysis (TLG). Tumor-related FDG uptake is considered pathologic if it exceeds 2.5. The highest uptake within the tumor is called SUVmax. SUVmean is the mean activity of the voxels within the volume of interest (VOI). Metabolic tumor volume (MTV) is defined as the total tumor volume contained in the VOI. Total lesion glycolysis (TLG) is calculated as MTV multiplied by the mean SUV of the lesion. For the MTV calculation, an SUV threshold of 2.5 or 40–42% of SUVmax can be used, as various examples in the response evaluation of tumor therapies show [9,10,11].

For analyzing TLG and MTV, we used the PET Edge [12,13,14] tool on MIM software for PET image analysis (version 7.1.4, MIM Software Inc., Cleveland, OH, USA). PET Edge uses its own algorithm with a gradient-based technique that detects the steepest drop in PET activity to create the contour boundary. This does not have an absolute or % change threshold but does not essentially differ from thresholds when an SUV value of 2.5 or 40% SUVmax is used [14]; even some researchers consider this gradient-based method preferable to assess volumetric tumor response in cancer treatment [14]. At our institution, this method is the current clinical routine.

Case 1: Our first patient was diagnosed with *BRAFV600* mutated non-small cell lung cancer (NSCLC) initially in 2014. The patient started treatment with vemurafenib, which he received for almost 6 years when he developed progressive disease [15]. Subsequently, his tumors progressed and were treated with an investigational RAF inhibitor and achieved disease control for about 1.5 years, but eventually, the tumor progressed, and with declining PS, the patient was elected for hospice.

**Figure 2:**
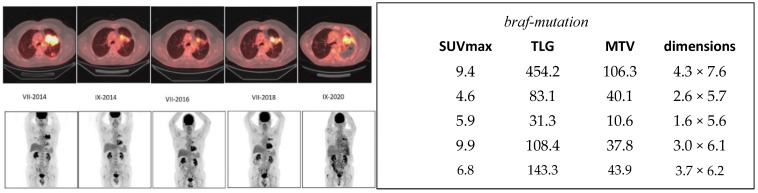
Consecutive ^18^F-FDG-PET/CT images in a non-small cell lung cancer patient who had a braf mutation and was treated with vemurafenib for almost 6 years. The upper panel on the left demonstrates PET/CT fusion images at the same level from July 2014 (VII-2014) to September 2020 (IX-2020). The lower panel on the left shows the MIP images during the same time period (VII-2014–IX-2020). On the right, the quantitative imaging is tabulated in the same chronological order. SUVmax, TLG, MTV, and tumor dimension values are presented in the beginning, i.e., the three first studies, and a response can be seen in SUV, TLG, and MTV values. The actual tumor size decreases, which can also be seen visually.

The serial imaging results are shown in Figure 2. At the baseline, in July 2014, an SUVmax of 9.4, total lesion glycolysis (TLG) of 454, and metabolic tumor volume (MTV) of 106 cm^3^ were used. The tumor size was 4.3 cm × 7.6 cm, as measured from CT. The tumor decreased in size until July 2016 with TLG at 31, MTV at 10.6 cm^3^, and the tumor size was 1.6 cm × 5.6 cm. All measurements (SUVmax, TLG, MTV, size) increased by the next PET study in July 2018, indicating a relapse. In the follow-up, during the next two years, the measurements slightly increased until September 2020 but were still below the numbers seen at the baseline. The quantitative imaging data are tabulated in Figure 2, including consecutive SUVmax, TLG, MTV, and tumor dimension values.

Case 2: The patient was diagnosed with RET fusion-positive NSCLC initially in 2018. The patient received treatment with a highly potent and selective RET inhibitor, selpercatninib, as part of a clinical trial and continued to derive clinical benefit [16].

**Figure 3:**
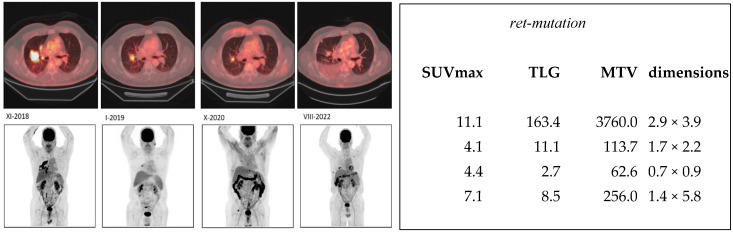
Consecutive ^18^F-FDG-PET/CT images in a non-small cell cancer patient who had a ret-mutation and was treated with selpercanitib in 2018. The upper panel on the left demonstrates PET/CT fusion images at the same level from November 2018 (XI-2018) to August 2022 (VIII-2022). The lower panel on the left shows the MIP images during the same time period (XI-2018–VIII-2022). On the right, the quantitative imaging is tabulated in the same chronological order. SUVmax, TLG, MTV, and tumor dimension values are presented at the beginning, i.e., the first three studies, where a response can be seen in SUV, TLG, and MTV values. The actual tumor size decreased, which can also be seen visually. A relapse can be seen visually and in quantitative imaging values, but the patient still experienced a clinical benefit from the treatment.

At the baseline, in November 2018, SUVmax was 11.1, total lesion glycolysis was 163, and the metabolic tumor volume was 3760. The tumor size was 2.9 cm × 3.9 cm as measured from CT. The tumor decreased in size until November 2020, with TLG at 11.1, MTV at 114, and the tumor size was 0.7 cm × 0.9 cm. All measurements (SUVmax, TLG, MTV, size) increased by the next PET study in August 2022, indicating a relapse (Figure 3). 

Case 3: The patient had a small intestine gastrointestinal stromal tumor (GIST), which was first diagnosed in 2000, as previously published [17]. The tumor was surgically removed. After three years, the patient had a disease relapse, which was treated with experimental peri-operative imatinib and was continued for another 2 years. After 2 years of imatinib being stopped, the patient had local recurrence and restarted imatinib, which she received for 4 years until she experienced disease progression. She was then started on sunitinib, a multi-kinase VEGF-based TKI, which controlled her disease for 2 more years with an interim surgical intervention. She then started regorafenib, another multi-kinase VEGF-based TKI, but rapidly progressed in the first restaging attempt. Similar results were obtained with sorafenib, a multi-kinase VEGF-based TKI afterward. Later, she started nilotinib, a c-KIT inhibitor that controlled her disease for 1 year before disease progression, and later pazopanib, another multi-kinase VEGF-based TKI. In 2016, she started a clinical trial with an investigational KIT inhibitor, which led to clinical benefit for 4 years with interim surgical resections. After progression, she received avapritinib, a selective KIT inhibitor, but eventually died after 4 months.

**Figure 4:**
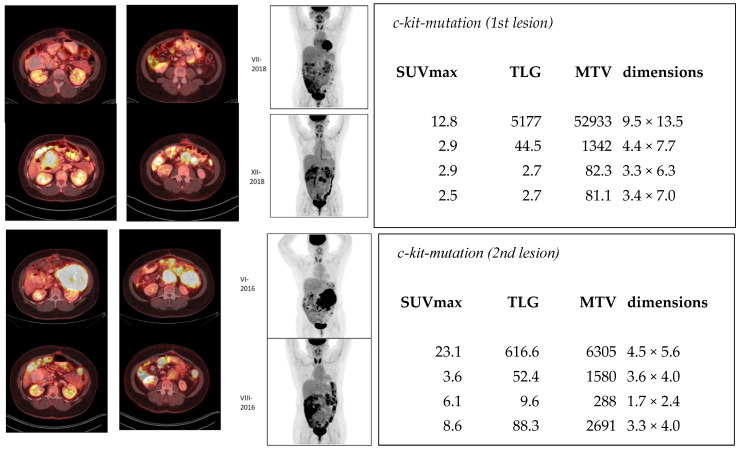
**[**^18^F]FDG-PET/CT images of a patient who had a small intestine gastrointestinal stromal tumor with a kit mutation and was treated with an investigational kit inhibitor from 2016. The upper panel on the left demonstrates PET/CT fusion images at two levels from June 2016 (VI-2016) to August 2018 (VIII-2018) and the corresponding MIP images during the same time period (VI-2016–VIII-2018). On the right, in the upper panel, the quantitative imaging is tabulated in the same chronological order. SUVmax, TLG, MTV, and tumor dimension values are presented; in this lesion, a response can be seen in SUV, TLG, and MTV values. The actual tumor size decreased, which can also be seen visually. The upper panel on the left demonstrates PET/CT fusion images at two levels from September (IX-2018) to December 2020 (XII-2020) and the corresponding MIP images during the same time period (IX-2018–XII-2020). There was a new lesion which increased in size. The quantitative imaging parameters increased in the last two studies, as seen on the right in the lower panel (adapted with different images for this figure from [17]).

The imaging results are shown in Figure 4. At the baseline, in June 2016, SUVmax was 12.8, total lesion glycolysis was 5177, and metabolic tumor volume was 52,933. The tumor size was 9.5 cm × 13.5 cm, as measured from CT. The tumor decreased in size until December 2020, where SUVmax was 2.5, TLG was 2.7, MTV was 81.1, and the tumor size was 3.4 cm × 7.0 cm. There was a recurrence in a different lesion, which was noticed in September 2018 and increased by December 2020, which is shown in the image. The characteristics of this second lesion are shown in Figure 4.

## 3. Discussion

Fluorodeoxyglucose (FDG) positron emission tomography (PET) imaging has long served as a cornerstone in oncological diagnostics, leveraging the heightened glucose metabolism characteristic of malignant cells. In the context of targeted therapies with TKIs, FDG uptake offers a dynamic glimpse into tumor biology, reflecting alterations in cellular metabolism induced by therapeutic interventions. By quantifying changes in FDG uptake pre- and post-treatment, clinicians can discern treatment response, anticipate disease progression, and tailor therapeutic regimens accordingly.

In this report, we present the cases of three patients who exhibited positive responses to targeted treatment aimed at specific mutations. Our findings indicate that molecular imaging provides more informative/qualitative insights into the patients’ conditions compared to morphological imaging, such as the tumor dimensions measured from CT scans. Specifically, the analysis of [^18^F] FDG-PET studies reveals that measurements of metabolic tumor volume (MTV) and total lesion glycolysis (TLG) offer more accurate information than maximum standardized uptake value (SUVmax) measurements. For instance, in the second patient (Figure 3), we observed a decrease in TLG, MTV, and tumor size over the course of the first three studies, while SUVmax showed a marginal increase from 4.1 to 4.4. Similarly, in our first patient (Figure 2), TLG, MTV, and tumor size decreased during the initial three studies, while SUVmax increased from 4.6 to 5.9. These findings highlight the importance of considering MTV and TLG measurements in conjunction with tumor size and SUVmax when evaluating treatment response using [^18^F]FDG-PET imaging.

The complexity of quantitative evaluation escalates when multiple lesions are involved, as illustrated by our third patient. Throughout the study period (VI-2016–XII-2020), it appears that the size of the initial lesion (Figure 4) decreased. However, a new lesion emerged just one month after the previous study (IX-2018), triggering suspicion.

For over two decades, experimental evidence has linked tyrosine kinase inhibitors to GLUT1 transport [1]. Studies on HL-60 cells and human erythrocytes revealed that these inhibitors impede the cellular uptake of deoxyglucose mediated by GLUT1, which is a multifunctional transporter responsible for hexose and dehydroascorbic acid transportation [18]. Various tyrosine kinase inhibitors, including tyrphostins, were found to inhibit deoxyglucose and dehydroascorbic acid transport in a dose-dependent manner [19]. Furthermore, ATP was found to regulate GLUT1 activity, with tyrosine kinase inhibitors completely blocking transport through this transporter. This inhibition’s competitive nature underscores the significance of putative nucleotide binding sites in GLUT1.

In another study, the interaction between tyrosine kinase inhibitors and human glucose transporter-1 (GLUT-1) was explored in FaDu and GIST-1 cells [2]. The uptake of hexose compounds was inhibited to varying degrees by these inhibitors, with representative inhibitors showing competitive inhibition. Interestingly, certain inhibitors irreversibly inhibited glucose uptake, raising questions regarding the reliability of [^18^F] FDG-PET uptake as a measure of tumor response.

However, findings from cell experiments could not be replicated in animal models. Mice with xenografts were treated with crizotinib, but the effects on tumor volume and [^18^F] FDG and [^18^F] FLT uptake varied. While crizotinib caused tumor regression in c-MET-amplified xenografts, the effect on [^18^F] FDG uptake differed between tumor types. Additionally, in vitro experiments with crizotinib showed inconsistent effects on glucose uptake but not on [^18^F] FLT.

In conclusion, while tyrosine kinase inhibitors show promise in inhibiting glucose uptake, translating these findings from cellular experiments to clinical applications presents challenges. The differential response observed in animal models emphasizes the need for comprehensive evaluation methods in assessing tumor response. In an animal tumor model, FLT emerged as a superior tracer compared to FDG, as reported by Cullinane et al. [1].

Recent studies have explored the utilization of labeled TKIs in patients. In particular, in non-small cell lung cancer (NSCLC), driven by activating epidermal growth factor receptor (EGFR) mutations, EGFR-TKIs have emerged as the optimal therapeutic approach [20]. Evaluating ^11^C-erlotinib, ^18^F-afatinib, and ^11^C-osimertinib for biodistribution and kinetics revealed intriguing findings. Among them, ^11^C-erlotinib and ^18^F-afatinib showcased the most promising tumor-to-background contrast in EGFR-positive lesions, while ^11^C-osimertinib exhibited the highest SUV and tumor-to-background ratio AUC values across various tissues [21].

Despite these advancements, FLT encounters similar challenges with tumor-to-background contrast in patients, as noted by Subbiah et al. [21]. Thus, FDG remains the preferred option.

Nevertheless, our findings indicate that inhibition by TKI inhibitors, such as BRAF, RET, and c-KIT inhibitors, does not significantly impact FDG follow-up. While FLT proves effective for early-response assessments in several cancer types, like lung cancer and sarcomas, it falls short in prostate cancer evaluations [22,23,24,25,26].

FDG data based on SUVmax TLG or MTV consistently correlated with RECIST data, providing valuable insights into tumor size and response assessment.

Preclinical studies have underscored the intricate interplay between TKIs and glucose metabolism pathways, elucidating mechanisms by which TKIs modulate FDG uptake in tumor cells. Notably, experimental evidence suggests that TKIs can impede glucose transporter activity, consequently attenuating FDG uptake in susceptible tumor types. Moreover, clinical investigations have corroborated these findings, demonstrating correlations between FDG PET parameters and treatment outcomes in patients undergoing TKI therapy. Such insights not only validate FDG uptake as a surrogate marker of treatment response but also highlight its potential utility in prognostication and therapeutic decision making.

Despite its promise, the integration of FDG PET imaging into routine clinical practice poses several challenges. Variability in FDG uptake patterns, tumor heterogeneity, and non-specific uptake in inflammatory or benign lesions necessitate the cautious interpretation of PET findings. Furthermore, the optimal timing and frequency of FDG PET assessments during TKI therapy remain subjects of ongoing debate. Addressing these challenges mandates concerted efforts to standardize imaging protocols, refine quantitative analyses, and validate FDG uptake as a robust biomarker across diverse tumor types and treatment regimens.

## 4. Conclusions

The assessment of treatment response in cancer patients undergoing targeted therapies with TKIs represents a multifaceted endeavor, wherein FDG uptake serves as a valuable adjunct to traditional imaging modalities. By unraveling the intricate relationship between TKIs and glucose metabolism, FDG PET imaging offers unique insights into therapeutic efficacy, disease progression, and patient outcomes. As we navigate the evolving landscape of precision oncology, harnessing the full potential of FDG uptake in response assessments promises enhancements in therapeutic decision making and the improvement of clinical outcomes for patients battling cancer.

## Data Availability

The original contributions presented in the study are included in the article material, further inquiries can be directed to the corresponding author.

## References

[1] Cullinane C., Dorow D.S., Jackson S., Solomon B., Bogatyreva E., Binns D., Young R., Arango M.E., Christensen J.G., McArthur G.A. (2011). Differential (18)F-FDG and 3′-deoxy-3′-(18)F-fluorothymidine PET responses to pharmacologic inhibition of the c-MET receptor in preclinical tumor models. J. Nucl. Med..

[2] Damaraju V.L., Aminpour M., Kuzma M., Winter P., Preto J., Tuszynski J., McEwan A.B.J., Sawyer M.B. (2021). Tyrosine kinase inhibitors reduce glucose uptake by binding to an exofacial site on hGLUT-1: Influence on ^18^ F-FDG PET uptake. Clin. Transl. Sci..

[3] Bijlstra O.D., Boreel M.M.E., van Mossel S., Burgmans M.C., Kapiteijn E.H.W., Oprea-Lager D.E., Rietbergen D.D.D., van Velden F.H.P., Vahrmeijer A.L., Swijnenburg R.-J. (2022). The Value of ^18^F-FDG-PET-CT Imaging in Treatment Evaluation of Colorectal Liver Metastases: A Systematic Review. Diagnostics.

[4] Ling T., Zhang L., Peng R., Yue C., Huang L. (2022). Prognostic value of ^18^F-FDG PET/CT in patients with advanced or metastatic non-small-cell lung cancer treated with immune checkpoint inhibitors: A systematic review and meta-analysis. Front. Immunol..

[5] Ko W.S., Kim S.J. (2023). Predictive Value of 18 F-FDG PET/CT for Assessment of Tumor Response to Neoadjuvant Chemotherapy in Bladder Cancer. Clin. Nucl. Med..

[6] Wang H.H., Steffens E.N., Kats-Ugurlu G., van Etten B., Burgerhof J.G.M., Hospers G.A.P., Plukker J.T.M. (2024). Potential Predictive Immune and Metabolic Biomarkers of Tumor Microenvironment Regarding Pathological and Clinical Response in Esophageal Cancer After Neoadjuvant Chemoradiotherapy: A Systematic Review. Ann. Surg. Oncol..

[7] Agostino N.M., Chinchilli V.M., Lynch C.J., Koszyk-Szewczyk A., Gingrich R., Sivik J., Drabick J.J. (2011). Effect of the tyrosine kinase inhibitors (sunitinib, sorafenib, dasatinib, and imatinib) on blood glucose levels in diabetic and nondiabetic patients in general clinical practice. J. Oncol. Pharm. Pract..

[8] Hadova K., Mesarosova L., Kralova E., Doka G., Krenek P., Klimas J. (2021). The tyrosine kinase inhibitor crizotinib influences blood glucose and mRNA expression of GLUT4 and PPARs in the heart of rats with experimental diabetes. Can. J. Physiol. Pharmacol..

[9] Usmanij E.A., de Geus-Oei L.F., Troost E.G., Peters-Bax L., van der Heijden E.H., Kaanders J.H., Oyen W.J., Schuurbiers O.C., Bussink J. (2013). ^18^F-FDG PET early response evaluation of locally advanced non-small cell lung cancer treated with concomitant chemoradiotherapy. J. Nucl. Med..

[10] Castello A., Rossi S., Lopci E. (2020). ^18^F-FDG PET/CT in Restaging and Evaluation of Response to Therapy in Lung Cancer: State of the Art. Curr. Radiopharm..

[11] Lopci E., Elia C., Catalfamo B., Burnelli R., De Re V., Mussolin L., Piccardo A., Cistaro A., Borsatti E., Zucchetta P. (2022). Prospective Evaluation of Different Methods for Volumetric Analysis on [^18^F]FDG PET/CT in Pediatric Hodgkin Lymphoma. J. Clin. Med..

[12] Mikell J.K., Kaza R.K., Roberson P.L., Younge K.C., Srinivasa R.N., Majdalany B.S., Cuneo K.C., Owen D., Devasia T., Schipper M.J. (2018). Impact of ^90^Y PET gradient-based tumor segmentation on voxel-level dosimetry in liver radioembolization. EJNMMI Phys..

[13] Guezennec C., Bourhis D., Orlhac F., Robin P., Corre J.B., Delcroix O., Gobel Y., Schick U., Salaün P.Y., Abgral R. (2019). Inter-observer and segmentation method variability of textural analysis in pre-therapeutic FDG PET/CT in head and neck cancer. PLoS ONE.

[14] Trada Y., Lin P., Lee M.T., Jameson M.G., Chlap P., Keall P., Moses D., Fowler A. (2023). Impact of tumour region of interest delineation method for mid-treatment FDG-PET response prediction in head and neck squamous cell carcinoma undergoing radiotherapy. Quant. Imaging Med. Surg..

[15] Subbiah V., Gervais R., Riely G., Hollebecque A., Blay J.Y., Felip E., Schuler M., Gonçalves A., Italiano A., Keedy V. (2019). Efficacy of Vemurafenib in Patients with Non-Small-Cell Lung Cancer with BRAF V600 Mutation: An Open-Label, Single-Arm Cohort of the Histology-Independent VE-BASKET Study. JCO Precis. Oncol..

[16] Drilon A., Oxnard G.R., Tan D.S., Loong H.H., Johnson M., Gainor J., McCoach C.E., Gautschi O., Besse B., Cho B.C. (2020). Efficacy of Selpercatinib in RET Fusion-Positive Non-Small-Cell Lung Cancer. N. Engl. J. Med..

[17] Gouda M.A., Janku F., Somaiah N., Hunt K.K., Yedururi S., Subbiah V. (2023). Multi-disciplinary management of recurrent gastrointestinal stromal tumor harboring KIT exon 11 mutation with the switch-control kinase inhibitor ripretinib and surgery. Oncoscience.

[18] Vera J.C., Reyes A.M., Velásquez F.V., Rivas C.I., Zhang R.H., Strobel P., Slebe J.C., Núñez-Alarcón J., Golde D.W. (2001). Direct inhibition of the hexose transporter GLUT1 by tyrosine kinase inhibitors. Biochemistry.

[19] Ohmichi M., Pang L., Ribon V., Gazit A., Levitzki A., Saltiel A.R. (1993). The tyrosine kinase inhibitor tyrphostin blocks the cellular actions of nerve growth factor. Biochemistry.

[20] van de Stadt E.A., Yaqub M., Schuit R.C., Bartelink I.H., Leeuwerik A.F., Schwarte L.A., de Langen A.J., Hendrikse H., Bahce I. (2022). Relationship between Biodistribution and Tracer Kinetics of ^11^C-Erlotinib,^18^F-Afatinib and ^11^C-Osimertinib and Image Quality Evaluation Using pharmacokinetic/Pharmacodynamic Analysis in Advanced Stage Non-Small Cell Lung Cancer Patients. Diagnostics.

[21] Subbiah V., Chuang H.H., Gambhire D., Kairemo K. (2017). Defining Clinical Response Criteria and Early Response Criteria for Precision Oncology: Current State-of-the-Art and Future Perspectives. Diagnostics.

[22] Kairemo K., Santos E.B., Macapinlac H.A., Subbiah V. (2020). Early Response Assessment to Targeted Therapy Using 3′-deoxy-3′[(18)F]-Fluorothymidine (^18^F-FLT) PET/CT in Lung Cancer. Diagnostics.

[23] Alwadani B., Dall’Angelo S., Fleming I.N. (2021). Clinical value of 3′-deoxy-3′-[^18^F]fluorothymidine-positron emission tomography for diagnosis, staging and assessing therapy response in lung cancer. Insights Imaging.

[24] Benz M.R., Czernin J., Allen-Auerbach M.S., Dry S.M., Sutthiruangwong P., Spick C., Radu C., Weber W.A., Tap W.D., Eilber F.C. (2012). 3′-deoxy-3′-[18F]fluorothymidine positron emission tomography for response assessment in soft tissue sarcoma: A pilot study to correlate imaging findings with tissue thymidine kinase 1 and Ki-67 activity and histopathologic response. Cancer.

[25] Kairemo K., Ravizzini G.C., Macapinlac H.A., Subbiah V. (2017). An Assessment of Early Response to Targeted Therapy via Molecular Imaging: A Pilot Study of 3′-deoxy-3′[(18)F]-Fluorothymidine Positron Emission Tomography ^18^F-FLT PET/CT in Prostate Adenocarcinoma. Diagnostics.

[26] Scarpelli M., Zahm C., Perlman S., McNeel D.G., Jeraj R., Liu G. (2019). FLT PET/CT imaging of metastatic prostate cancer patients treated with pTVG-HP DNA vaccine and pembrolizumab. J. Immunother. Cancer.

